# Size-driven parr-smolt transformation in masu salmon (*Oncorhynchus masou*)

**DOI:** 10.1038/s41598-023-43632-7

**Published:** 2023-10-03

**Authors:** Yuki Ugachi, Haruka Kitade, Eisuke Takahashi, Shotaro Suzuki, Mizuki Hayashi, Taiga Yamada, Wenda Cui, Munetaka Shimizu

**Affiliations:** 1 School of Fisheries Sciences, Hokkaido University, 3-1-1 Minato, Hakodate, Hokkaido 041-8611 Japan; 2 Graduate School of Environmental Science, Hokkaido University, Kita 10, Nishi 5, Kita-ku, Sapporo, Hokkaido 060-0810 Japan; 3 Nanae Fresh-Water Station, Field Science Center Northern Biosphere, Hokkaido University, 2-9-1 Sakura, Nanae, Kameda-gun, Hokkaido 041-1105 Japan; 4 Faculty of Fisheries Sciences, Hokkaido University, 3-1-1 Minato, Hakodate, Hokkaido 041-8611 Japan; 5 Field Science Center for Northern Biosphere, Hokkaido University, 3-1-1, Hakodate, Hokkaido 041-8611 Japan

**Keywords:** Animal physiology, Animal migration, Marine biology

## Abstract

Anadromous salmonids exhibit partial migration, where some individuals within a population migrate down to the ocean through complex interactions between body size and photoperiod. This study aimed to integrate the ontogenetic and seasonal patterns of smoltification, a series of changes for future marine life, in a strain of masu salmon (*Oncorhynchus masou*). Spring smoltification, as evidenced by the activation of gill Na^+^,K^+^-ATPase (NKA), was induced during winter under an advanced photoperiod. In addition, juveniles showed an additional peak in gill NKA activity in August regardless of the photoperiod. When juvenile masu salmon were subjected to feeding manipulations during the first spring/summer, only fish exceeding a fork length of 12 cm exhibited an increased gill NKA activity. We tested whether size-driven smoltification required a long-day period by exposing juveniles to a constant short-day length (9-h light and 15-h dark) from January to November. Juveniles under short-day conditions exceeded 12 cm in June but showed no signs of smoltification. Thus, masu salmon undergo photoperiod-limited, size-driven smoltification during the first summer and size-limited, photoperiod-driven smoltification the following spring. The findings of the present study provide a framework for further elucidation of the physiological mechanisms underlying partial migration in salmonids.

## Introduction

Migration is a patterned movement between breeding and non-breeding sites that is observed in a wide range of animal taxa, including birds, fish, reptiles, mammals, insects, and crustaceans^1–3^. The ultimate considerations for migration are maximising the individual fitness and stability of populations/species. However, in certain species and situations, variations exist within a population, where some members prepare and commence migration while others do not^4, 5^. This divergence in migration patterns within a population or species is known as partial migration. Partial migration is conditional to individual state, and there are threshold value(s)/conditions to decide upon migration^4, 5^.

Several species of salmonids, such as the Atlantic salmon (*Salmo salar*), masu salmon (*Oncorhynchus masou*), Chinook salmon (*O. tshawytscha*), and brown trout (*S. trutta*), exhibit partial anadromous migration^6–9^. Juvenile salmon prepare for future marine life through a series of physiological, morphological, and behavioural changes called parr-smolt transformation (smoltification). During smoltification, river-dwelling parr acquire hypo-osmoregulatory ability that is accompanied with increased Na^+^,K^+^-ATPase (NKA) activity in the gills; become silvery, large, and slim; and down-migrate to the ocean as ocean-type smolts^10–13^. Smoltification of salmonids is a developmental and seasonal event that typically occurs in the second spring of life while it is also seen in autumn in some strains of Chinook salmon and masu salmon^7, 14^. Environmental factors such as photoperiod and water temperature affect the timing and degree of smoltification^15^. In Atlantic salmon, photoperiod is the primary factor that synchronises the timing of spring smoltification whereas water temperature does not act as a zeitgeber but influences the speed and degree of spring smoltification^16, 17^.

The decision to commence smoltification and down-migrate to the ocean in spring is made in previous autumn through a complex interaction among the developmental stage (age), growth opportunities, and environmental cues^18, 19^. In juvenile Atlantic salmon, individuals exceeding a certain size under decreasing day length during autumn are recruited as candidates for smolts while those not reaching the size threshold reside in the river as parr the following spring^6, 20^. Similar patterns have been observed in other salmonids^7, 9^. Thus, spring smoltification of salmonids is limited to the size and photoperiod-driven. However, there are several cases that do not fit this developmental pathway, where smoltification-like changes are often observed independent of the season, but are related to body size^21–25^, suggesting the presence of other developmental pathways in smoltification of salmonids.

Masu salmon (*O. masou*) is one of the eight Pacific salmon species distributed on the Asian side of Pacific Ocean, ranging from the north to the Amur River basin, the western side of Kamchatka Peninsula, and south of Taiwan Island^26^. This species is a good model to unravel the ontogenetic and proximate mechanisms of commencement of smoltification because its life-history patterns are complex enough to exhibit partial migration but relatively simple and semelparous with a typical lifespan of 3 years. Masu salmon generally stay in the river at high latitudes for one and a half year before undergoing smoltification in the spring, spend 1 year in the ocean, and return to freshwater for spawning^7^.

A square-wave photoperiod, where long days are disrupted by a period of short days, is routinary used to advance the time of spring smoltification in aquaculture industry^27–30^. We previously confirmed that this protocol induces spring smoltification in masu salmon^31^. In this study, we conducted a series of rearing experiments by manipulating photoperiod and feeding rations and found that there was a second pathway for the commencement of smoltification in masu salmon, a photoperiod-limited, size-driven smoltification occurring in the first summer in addition to the size-limited, photoperiod-driven smoltification in the following spring. The findings of the present study provide a framework for further elucidation of the physiological mechanisms underlying partial migration in salmonids.

## Results

### Effect of photoperiod on spring smoltification

**Figure 1:**
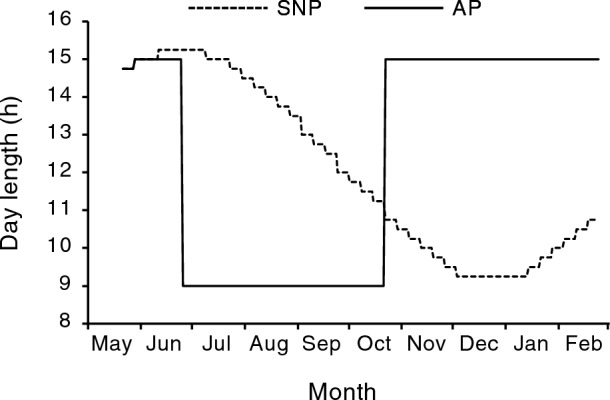
Two photoperiod regimes employed in the present study. A simulated natural photoperiod (SNP) was controlled by a timer and adjusted weekly to correspond with a latitude of N 41° 46′. An advanced photoperiod (AP) treatment started on June 22 by decreasing the photoperiod from a LD15:9 long-day length to a LD9:15 short-day length for 4 months and followed by the return to the long-day length on 23 October.

**Figure 2:**
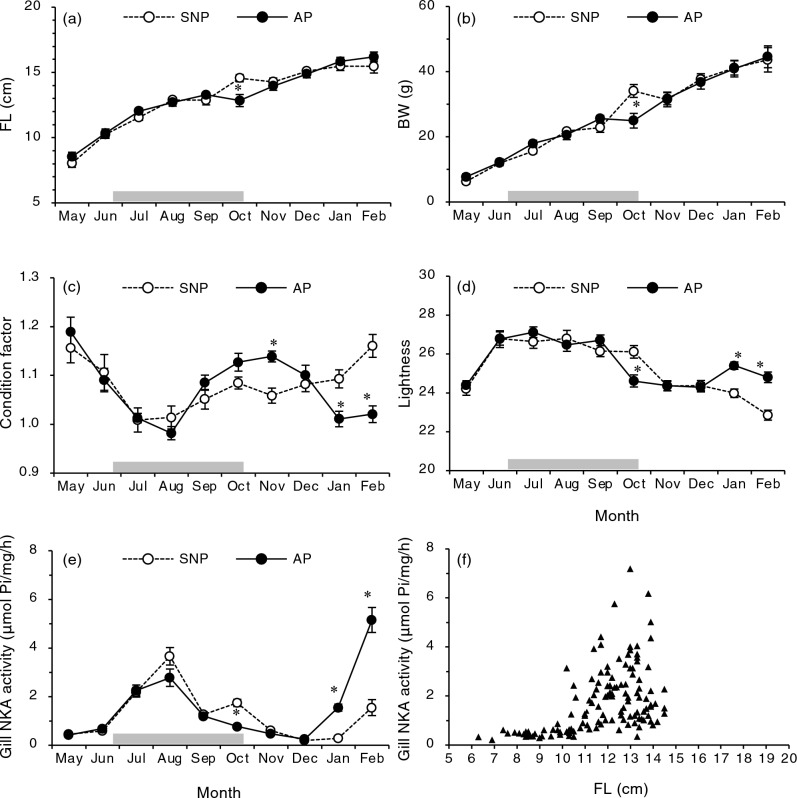
Effects of a square-wave photoperiod treatment on fork length (FL; **a**), body weight (BW; **b**), condition factor (**c**), body colour/lightness (**d**), gill Na^+^,K^+^-ATPase (NKA) activity (**e**) and relationship between FL and gill NKA activity (**f**) in juvenile masu salmon of Shiribetsu strain. Values are expressed as mean ± SE (*n* = 8–16). Data from precociously maturing males were not included. Asterisks indicate significant differences between photoperiod treatments in a given month (Student’s *t*-test, *P* < 0.05).

When the Shiribetsu fish were exposed to a square-wave photoperiod (Fig. 1), they grew similar to the control group (Fig. 2a,b) but exhibited morphological changes associated with smoltification, such as reduction in condition factor and body slivering, 3 months earlier than the regular timing (i.e. May) (Fig. 2c,d). In addition, AP fish showed a large increase in gill NKA activity, an index of hypo-osmoregulatory ability, in February (Fig. 2e). On the other hand, there was an additional peak in gill NKA activity in the Shiribetsu strain in August, regardless of the photoperiod (Fig. 2e). When gill NKA activity in the Shiribetsu strain was plotted against fork length from May to September, fish larger than 10–12 cm tended to have a higher activity (Fig. 2f).

### Effect of food restriction on summer smoltification

**Figure 3:**
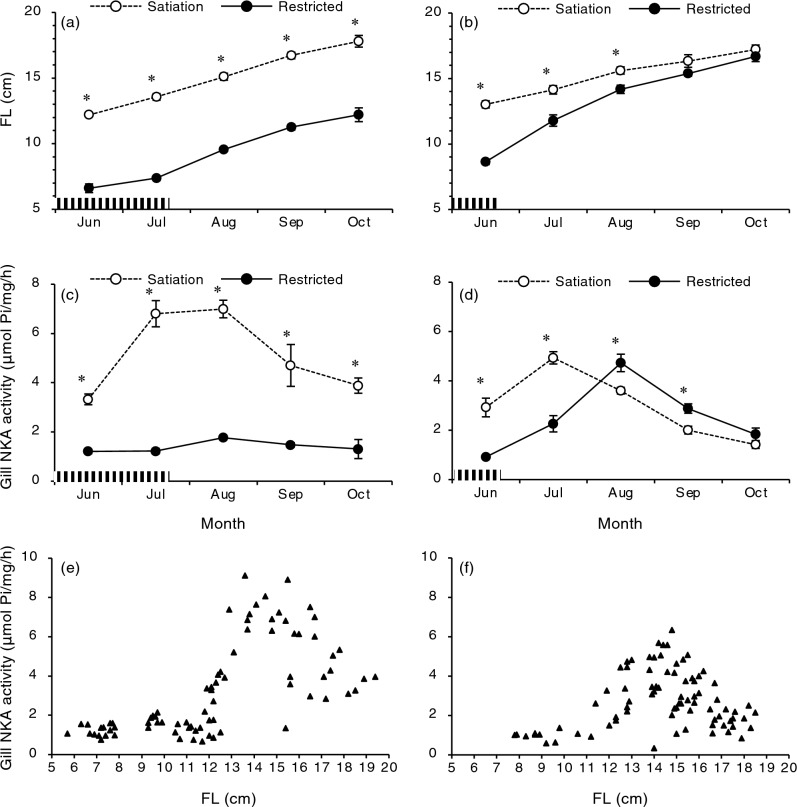
Effects of feeding restriction on fork length (FL; **a**,**b**), gill Na^+^,K^+^-ATPase (NKA) activity (**c**,**d**) and their relationship (**e**,**f**). Juvenile masu salmon of Shiribetsu strain were subjected to feeding restriction at 17% of a full ration during May to July (left) or 50% of a full ration during May to June (right). Values are expressed as mean ± SE (*n* = 7–8). Data from precociously maturing males were not included. Striped bars indicate the periods of food restriction. Asterisks indicate significant differences between photoperiod treatments in a given month (Student’s *t*-test, *P* < 0.05).

**Figure 4:**
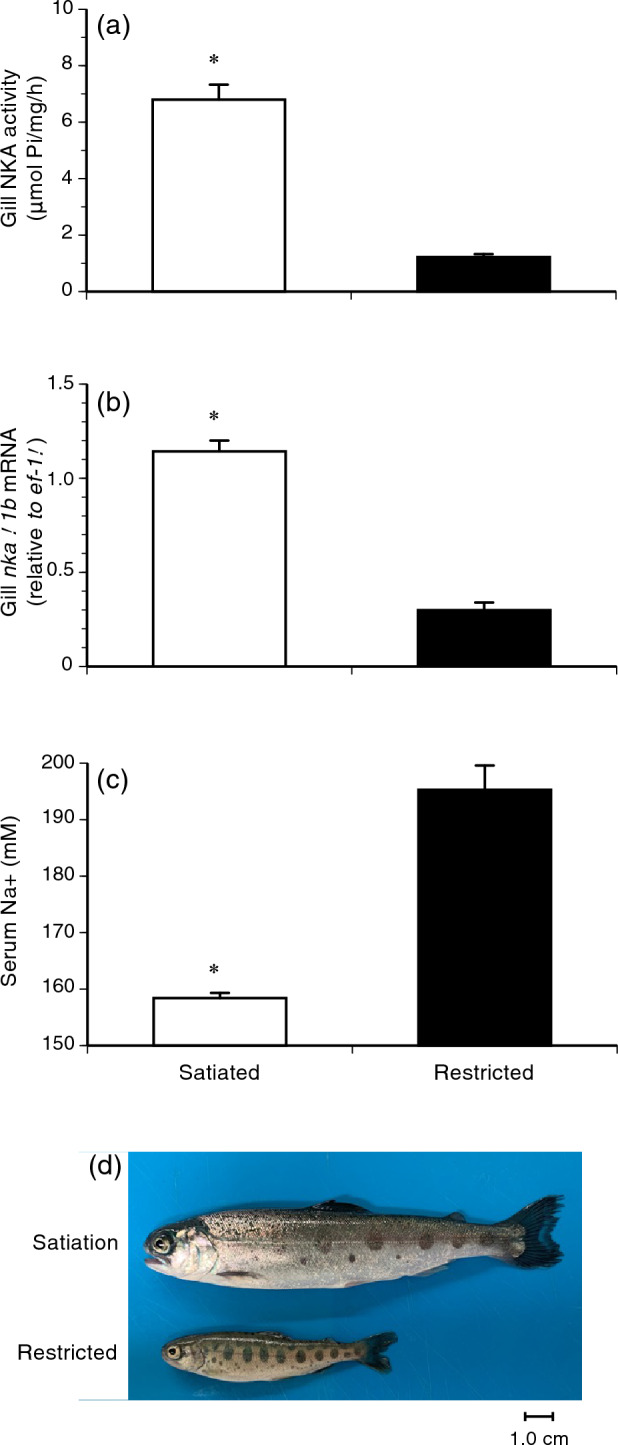
Comparisons of gill Na^+^,K^+^-ATPase (NKA) activity (**a**), gill *nka-α1b* mRNA levels (**b**), serum sodium ion levels 48 h after seawater transfer (**c**) and physical appearance (**d**) between fish fed for satiation and with restricted ration at 17%. Values are expressed as mean ± SE (*n* = 7–8). Data from precociously maturing males were not included. Asterisks indicate significant differences between feeding treatments in July (Student’s *t*-test, *P* < 0.05).

To test whether the increase in gill NKA activity during summer was driven by body size, juveniles of the Shiribetsu strain were subjected to food restriction in another set of experiments (Fig. 3). In the first trial, the satiety-fed control group showed an increase in gill NKA activity when individuals exceeded a fork length of 12 cm (Fig. 3a,c,e). In contrast, a group fed one-sixth of the full ration from May to July did not exceed a fork length of 12 cm nor showed an increase in gill NKA activity (Fig. 3a,c,e). In the second trial, one group fed half the full ration from May to June was able to catch up to the body size of control fish, which exceeded 12 cm in July (Fig. 3b). Both control and restricted fish showed a peak in gill NKA activity, but at different times in July and August, respectively (Fig. 3d), when individuals exceeded a length of 12 cm regardless of the treatment (Fig. 3f). In the first trial, increased gill NKA activity in fish fed at satiation was accompanied with increased mRNA levels of gill NKA α1b subunit, a seawater-type of α-subunit in salmonids, and lower serum sodium ion levels after 48 h of transfer to 70% seawater (i.e., 23 psu) (Fig. 4a–c). Moreover, well-fed fish exhibited a silver body colour characteristic of smolts (Fig. 4d). These results indicated that summer smoltification was induced by body size.

### Effect of short daylength on summer smoltification

**Figure 5:**
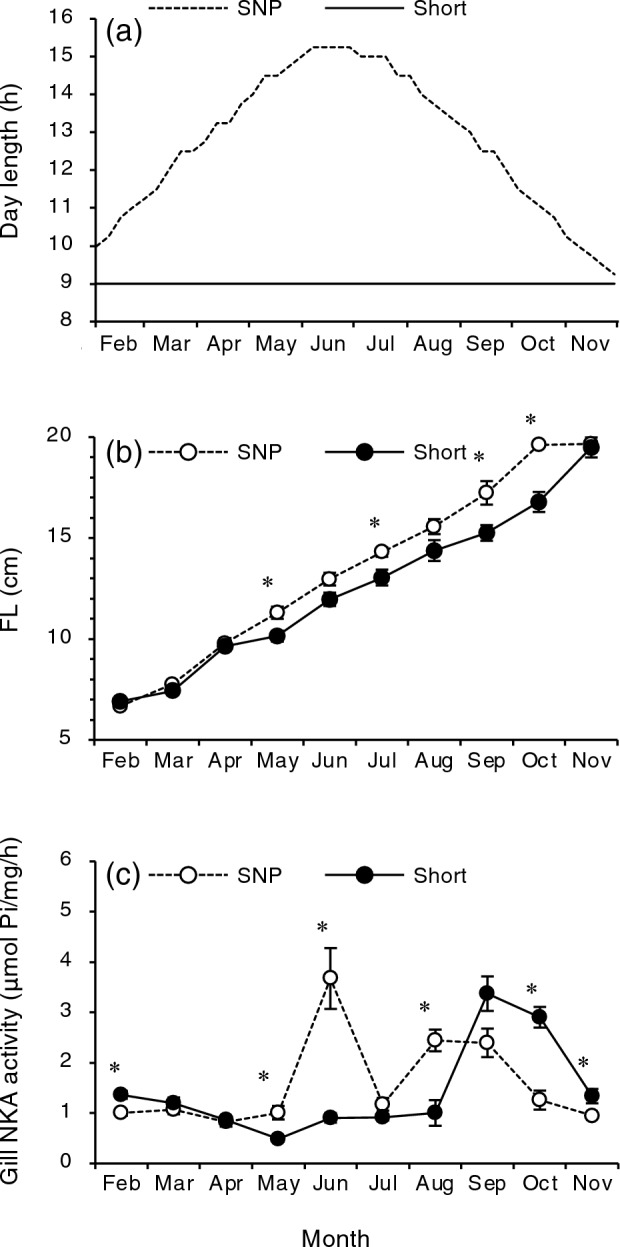
Effects of a constant short daylength after first feeding (**a**) on fork length (FL; **b**) and gill Na^+^,K^+^-ATPase (NKA) activity (**c**) of juvenile masu salmon of Shiribetsu strain. A simulated natural photoperiod (SNP) was controlled by a timer and adjusted weekly to that in the latitude at N 41° 46′. A short-day treatment (Short) started in January by setting daylength at LD9:15. Values are expressed as mean ± SE (*n* = 8). Asterisks indicate significant differences between photoperiod treatments in a given month (Student’s *t*-test, *P* < 0.05).

**Figure 6:**
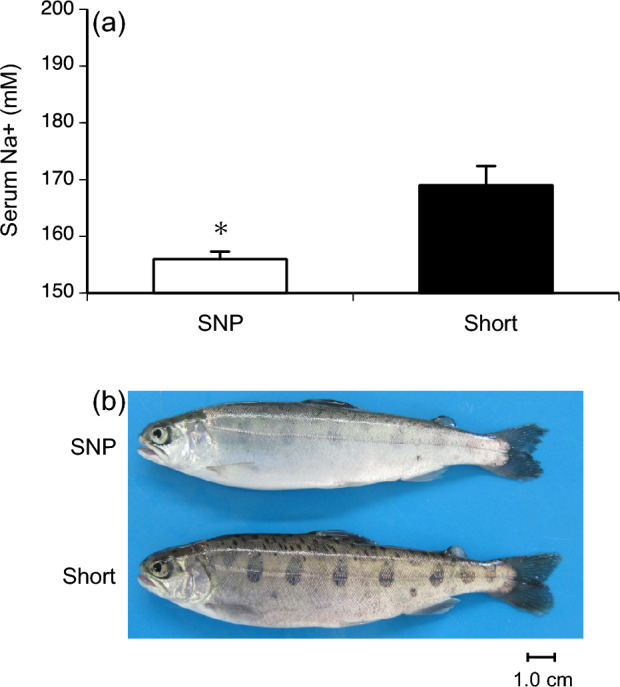
Comparisons of serum sodium ion levels 48 h after sweater transfer (**a**) and physical appearance (**b**) between fish under a natural photoperiod (SNP) and constant short daylength at LD9:15 (Short). Values are expressed as mean ± SE (*n* = 8). Asterisks indicate significant differences between photoperiod treatments in July (Student’s *t*-test, *P* < 0.05).

The impact of photoperiod on size-driven smoltification was studied during the first winter/spring. Juveniles after first feeding (fry) of Shiribetsu strains were maintained under a constant short daylength (9L:15D) from January to November (Fig. 5a). Although control fish under SNP grew slightly better than fish under short day lengths, both fish exceeded a length of 12 cm in June (Fig. 5b). However, the gill NKA activity increased only in the control group in June (Fig. 5c). Additionally, fish under SNP had lower serum sodium ion concentrations 48 h of transfer to 70% seawater and exhibited a silverly body colour compared to those under short day length (Fig. 6a,b). On the other hand, in the short-day group, an increase in gill NKA activity was observed in September (Fig. 5c), 3 months after the control fish increased their activity when they exceeded a 14-cm length (Data not shown). These results indicated that summer smoltification was photoperiod-limited.

## Discussion

A series of rearing experiments using masu salmon identified another developmental pathway for the commencement of size-driven but photoperiod-limited smoltification. This contrasts with the well-known size-limited and photoperiod-driven pathway. The first experiment with photoperiod manipulation during summer and autumn suggested a smoltification-like change independent of the photoperiod. The second set of experiments with food restrictions revealed that it was size driven. The third experiment, with manipulation of early photoperiod (i.e. winter and spring), indicated that size-driven smoltification was limited by the photoperiod. The findings of the present study provided an ontogenetic and seasonal framework to further unravel the physiological mechanisms underlying the decision to commence partial migration in salmonids.

Smoltification is a photoperiod-driven seasonal event in juvenile anadromous salmonids that generally occurs during spring^15^. In the present study, spring smoltification was induced by a square-wave photoperiod in Shiribetsu strain of masu salmon. In addition, a peak in gill NKA activity was observed in August, which was independent of the photoperiod. Moreover, juvenile masu salmon showed an increased gill NKA activity when its body length exceeded 12 cm. These result suggested that in addition to photoperiod-driven spring smoltification, size-driven smoltification occurred in the first summer of life. The importance of size in development of hypoosmoregulatory ability has been reported in Atlantic salmon, coho salmon, Chinook salmon, and rainbow trout (*O. mykiss*)^14, 25, 32, 33^. However, the ontogenetic relationship between body size and photoperiod with respect to smoltification remains largely unknown.

Food restriction in juveniles revealed that summer smoltification was driven by body size within a size threshold. In the two experiments, the satiated feeding groups exceeded a 12-cm length from June and showed a peak in gill NKA activity during July–August. The plot of gill NKA activity against body length showed that 12 cm was the threshold size to increase gill NKA activity. Decreased values of gill NKA in fish larger than 12 cm are most likely due to desmoltification, and a reverse change in physiological characteristics has been observed in fish kept in freshwater beyond the smoltification period^10, 11^. Food restriction to 17% of the full ration prevented juveniles from reaching a size of 12 cm until October and increasing the gill NKA activity. On the other hand, juveniles with moderate food restriction at 50% of the full ration exceeded a size of 12 cm in July and exhibited a peak in gill NKA in August. These results demonstrated that summer smoltification in masu salmon was indeed size-driven under a natural photoperiod and a fork length of 12 cm was the threshold size under the current experimental settings.

The present study also suggested a window for size-driven smoltification because the restricted feeding group reached a length of 12 cm in October but did not increase gill NKA activity. Decreasing photoperiod influences the smoltification window. Given that autumn is the time for decisions regarding spring smoltification^6, 7, 34^, the restricted group presumably entered the developmental pathway for spring smoltification.

However, it is possible that the commencement of size-driven summer smoltification was influenced by the photoperiod before summer solstice in late June. Clarke et al.^35^ reported that a short day followed by a long day during the first feeding in winter was required to induce underyearling smolting. The present study tested whether size-driven summer smoltification required a period of long days by exposing first-feeding juvenile masu salmon to a constant short-day length (9 L:15D) from January to November with a full ration while another group was reared under a simulated natural photoperiod. Although the control fish grew better than those under short day lengths, both groups exceeded a length of 12 cm in June. However, only control fish under natural photoperiod smoltified and fish under short day length showed no signs of smoltification in June, such as increased gill NKA activity and silverly body colour. These results supported the hypothesis that size-driven summer smoltification was influenced by a period of long days; thus, the photoperiod was a limiting factor.

Despite the failure of treated fish to commence summer smoltification, they exhibited a delayed peak in gill NKA activity in September. Thus, photoperiod manipulation did not completely inhibit size-driven smoltification. Moreover, under a constant short-day length, the threshold size for commencement of smoltification was approximately 14 cm, which was larger than 12 cm in the case of a natural photoperiod. The mechanism or significance of size difference should be explored in future studies. Steelhead, an anadromous form of rainbow trout, can initiate smoltification in the dark^25^, suggesting an endogenous rhythm for smoltification. The finding of the present study suggested that masu salmon was capable of commencing photoperiod-independent smoltification.

Our findings suggested that masu salmon was capable of undergoing size-driven smoltifcation if they experienced a period of long days. It is worth emphasising that the interaction between the photoperiod and status of juvenile fish around the first feeding was far more complex. In coho salmon, exposing underyearling juveniles to a long daylength (14.5L:8.5D) during February–April followed by a natural photoperiod prevents them from smolting in summer while the effect of exposure to short day length induces smoltification^35^, suggesting that juveniles need to grow or hatch early enough to respond to an increasing photoperiod and commence size-driven smoltification. This may be analogous to spring smoltification in yearling fish. However, the period of summer smoltification is not synchronised by the photoperiod but is broad depending on the individual growth status. The rearing protocol employed in the present study should aid in identifying the threshold size and time window for summer smoltification.

## Conclusion and perspective

**Figure 7:**
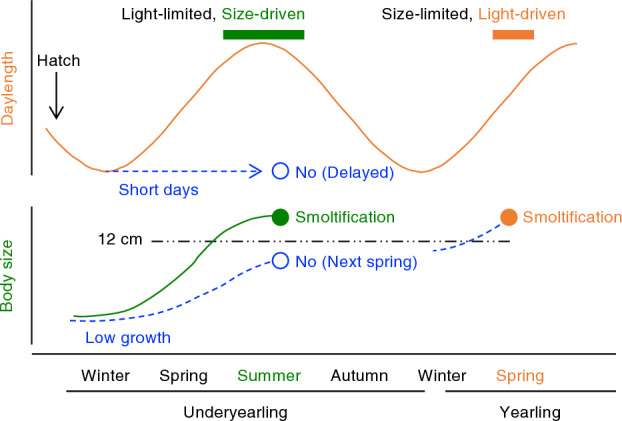
An ontogenetic and seasonal framework of two pathways for the commencement of smoltification in masu salmon.

The present study showed that masu salmon underwent photoperiod-limited, size-driven smoltification during the first summer and size-limited, photoperiod-driven smoltification the following spring. This ontogenetic framework is useful for a better understanding of the physiological consequences and mechanisms of decision, initiation, and completion of smoltification in salmonids (Fig. 7). There are many studies dealing with physiological changes during spring smoltification^10–13^. An increase in day length in spring drives the secretion of hormones such as growth hormone, insulin-like growth factor-1, cortisol, and thyroid hormones in fish exceeding a threshold size while non-candidates remain relatively inactive^36, 37^. However, little is known about the physiological basis of smoltification decisions made during previous autumn. Moreover, physiological studies on smoltification are difficult because the cause and effect influence each other and are difficult to disentangle. For instance, size is critical for the commencement of spring smoltification, but once it is decided, the growth rate is altered by changes in the metabolic rate^18, 19, 38^. Thus, identifying the developmental pathway, factors involved, and timing of smoltification decisions is important. The present study identified a second pathway for commencement of smoltification, in which the initial photoperiod was a limiting factor in the decision to smoltify and body size was a driver for initiation of smoltification. Although size-dependent smoltification has been reported previously^14, 25, 32, 33^, there are limited theories on such size-dependent changes and their relationship with photoperiod-driven spring smoltification^18, 39, 40^. The present study provided an integrated framework for unravelling the ontogenetic and proximate mechanisms of smoltification commencement and partial migration in salmonids.

## Materials and methods

### Experimental fish

The present study used a strain of masu salmon, Shiribetsu strain, from Hokkaido, Northern Japan. This strain is a captive brood stock originally obtained from the Shiribetsu River, Southwest Hokkaido, Japan, which has been maintained at Nanae Fresh-Water Laboratory, Field Science Center for Northern Biosphere, Hokkaido University (41° 90′ N; Kameda-gun, Hokkaido, Japan). Eggs and sperm of the Shiribetsu masu salmon were fertilised in mid-September, and fry were first fed in late November and reared under a natural photoperiod in 30-cm circular tanks (23 L) supplied with flow-through well-water (10 °C). Fish were fed a commercial diet (Marubeni Nisshin Feed Co., Ltd., Tokyo, Japan) daily for satiation.

### Effect of photoperiod on spring smoltification

A square-wave photoperiod manipulation used by Suzuki et al.^31^ was employed to the underyearlings of Shiribetsu strain in 2019 (Fig. 1A). In late April, 440 fish were equally divided in four 0.47-m circular tanks (50 L) supplied with flow-through well-water (10 °C) at 5 L/min, and both tanks were placed in a compartment shielded with black vinyl cloth. The photoperiod of each compartment was controlled using an LED light (810 lm; Iris Oyama, Miyagi, Japan) installed 30 cm above the tank. After 3 weeks of acclimation under a natural photoperiod with feeding, the tanks were assigned to one of the two photoperiod regimes, a simulated natural photoperiod (SNP), which was set for the latitude in Hakodate, Hokkaido, Japan (N41°46'), and an advanced photoperiod (AP), in which a LD15:9 long-day length was interrupted by a LD9:15 short-day length from late June to late October for 4 months (Fig. 1). Each photoperiod treatment was performed in duplicate. The fish were reared until mid-February 2019 under these conditions. They were fed once a day with the same diet as that used for satiation. Water temperatures were maintained around 10 °C throughout the experiment.

In May and June 2019, before the start of photoperiod manipulation, four fish from each of the four tanks (i.e., eight fish/treatment) were sampled each month. From July to February, eight fish from each of the four tanks (16 fish/ treatment) were sampled monthly. In June and October, fish in the AP group were sampled prior to the photoperiod. At each sampling point, the fish were anaesthetised with 3.3% 2-phenoxyethanol (Kanto Chemical, Tokyo, Japan). The fork length and body weight were measured, and the condition factor was calculated as follows: (body weight (g)) × 100/(fork length (cm))^3^. The lightness (L*) of the body colour was measured using a hand-held chromameter (CR-100; Konica Minolta Sensing, Osaka, Japan) as described in Suzuki et al.^31^ and used to evaluate the degree of body silvering. The appearance of precociously maturing males was determined by visual inspection of the developing testes or sperm, which were removed from the analyses. Gill filaments from the first arch were collected, immediately frozen on dry ice, and stored at − 80 °C until use.

### Effect of food restriction on summer smoltification

Two similar experiments with different degrees of food restriction were conducted on Shiribetsu underyearlings in 2021 and 2022. In 2021, 120 fry of Shiribetsu strain were reared under natural photoperiod in 30-cm circular tanks (23 L) supplied with flow-through well-water (10 °C) until March 2021, and they were then transferred to two circular 80-L tanks. Juveniles in one tank were fed daily until satiation in March and October. Juveniles in the other tank were fed half the amount of satiation twice a week during March and mid-July, and they were fed to satiation from mid-July to October. Fish were sampled monthly as described above.

In July, eight fish from each treatment group were transferred to 70% artificial seawater (23 practical salinity unit, psu; Napqo, Tokyo, Japan) in two 35-L square glass tanks (45 × 30 × 30 cm; Gex, Osaka, Japan). Salinity was monitored using a salinity meter (MotherTool, Nagano, Japan). Water temperature was maintained at 10 °C by placing the experimental tanks in a larger tank filled with running well-water. Food was withheld during the transfer. The fish were sampled after 48 h of transfer. Blood was collected from the caudal vein using a syringe or a plain 75-μL glass tube (Hirschmann, Eberstadt, Germany) after cutting the tail, and it was left to clot at 4 °C overnight. Serum was collected after centrifugation at 9730×*g* for 10 min and stored at − 80 °C until use.

In 2022, 120 fry of the Shiribetsu strain were subjected to another food-restriction experiment. Juveniles in one tank were fed daily to satiation during March and October whereas those in the other tank were fed daily at half-ration during March and mid-June followed by satiation until October. Fish were sampled monthly as described above.

### Effect of short daylength on summer smoltification

In January 2022, 160 fry of Shiribetsu strain were equally placed in four 50-L tanks supplied with flow-through well-water (10 °C) at 5 L/min, and both tanks were placed in a compartment shielded with black vinyl cloth. They were assigned to one of the two photoperiod treatments, SNP or constant short-day length (LD9:15). The photoperiod of the compartment was controlled using an LED light as described above. SNP was set for the latitude in Hakodate, Hokkaido, Japan (N 41° 46′). Animals were fed daily until satiation, reared, and sampled monthly until November. In July, eight fish from each treatment group were transferred to 70% artificial seawater as described above.

### NKA activity assay

Gill NKA activity was measured according to Quabius et al.^41^ with a minor modification. Protein concentration was measured using a bicinchoninic acid Protein Assay Kit (Thermo Scientific, IL, USA). Enzymatic activity was expressed as Pi (µmol) per mg protein per h.

### Serum ion levels

Twenty-five microlitres of serum diluted with an equal volume of water were applied to Fuji Dry-Chem Slide Na–K–Cl (Fuji Medical Co., Tokyo, Japan) to measure sodium and chloride concentrations using Fuji Dry Chem 4000 V (Fuji Medical Co., Tokyo, Japan).

### Reverse-transcribed quantitative polymerase chain reaction (RT-qPCR)

The abundance of gill *nka-α1b* mRNA, a seawater-type NKA α subunit increased during smoltification^42, 43^, in July was quantified via RT-qPCR as previously described in Suzuki et al.^31^. Briefly, total RNA was isolated from the gill samples and 1.5 μg was reverse-transcribed using oligo(dT_20_) primer and the Superscript III kit (Thermo Fisher Scientific, Waltham, MA, USA). A PCR mixture was set up using Power SYBR Green PCR Master Mix (Applied Biosystems, Carlsbad, CA, USA) with a reaction volume of 20 μL and primer concentration of 100 nM. Primer sequences for *nka-αlb* and *ef1α*^31^ are as follows. *nka-αlb* forward: gtacatttcaaccaacaacattacac, reverse: tagtgcaccatcacagtgttcat; *ef1α* forward: gaatcggccatgcccggtgac, reverse: ggatgatgacctgagcggtg. RT-qPCR was performed in a 7300 Sequence Detector (Applied Biosystems) with the following PCR conditions: 50 °C for 2 min, 95 °C for 10 min, 40 cycles of 15 s at 95 °C, and 60 °C for 1 min. The relative abundance of mRNA was normalised using *ef1α* as an endogenous reference gene.

### Statistical analysis

Statistical tests were performed using JMP software (SAS Institute Inc., Cary, NC, USA). Data from precociously maturing males were excluded from the analyses. The results of morphological and physiological parameters in the photoperiod treatments were first analysed using repeated-measures analysis (Mixed Model) by designating replicated tanks as experimental units. When significant effects were found, differences between months within the same treatment and between treatments within the same month were further identified using Tukey’s honest significant difference test and Student’s *t*-test, respectively. *P* < 0.05 was considered significant. The results of morphological and physiological parameters between food restrictions were analysed using Student’s *t*-test for each month. Results of gill NKA activity, gill *nka-α1b* mRNA abundance, and serum sodium ion concentration after seawater transfer were analysed using Student’s *t*-test.

### Ethical statement

All rearing protocols and experiments were approved by the Hokkaido University Animal Care and Use Committee (Approval No. 30-3) and performed in accordance with the guidelines and regulations. The present study was reported in accordance with ARRIVE guidelines.

## Data Availability

The datasets used and/or analysed during the current study are available from the corresponding author on reasonable request.
